# The nucleoid as a site of rRNA processing and ribosome assembly

**DOI:** 10.3389/fpls.2014.00257

**Published:** 2014-06-05

**Authors:** Alexandra-Viola Bohne

**Affiliations:** Department of Molecular Plant Sciences, Ludwig-Maximilians-University MunichPlanegg-Martinsried, Germany

**Keywords:** rRNA processing, ribosome assembly, nucleoid, plastid, mitochondria

Protein biosynthesis is one of the key elements of gene expression in living cells. All proteins are synthesized on ribonucleoprotein complexes, the ribosomes. In bacteria and their derivatives—eukaryotic mitochondria and plastids—ribosomes consist of a small and a large subunit which together comprise more than 50 ribosomal proteins and usually two to four ribosomal RNAs (rRNAs). Synthesis of the required rRNAs and proteins, and their correct folding, maturation/modification and assembly into functional particles are highly coordinated. However, whereas ribosomal composition and many mechanistic aspects of their biogenesis are well understood, little is known about the spatial organization of the procedure in bacteria and organelles. In eukaryotes, the individual processes involved occur in defined regions of the cell: ribosomal proteins are synthesized in the cytosol, but most rRNAs are transcribed, processed and modified in the nucleolus, a distinct subnuclear compartment (Lafontaine and Tollervey, 2001; Boisvert et al., 2007). Only the transcription of the small *5S* rRNA occurs in the nucleoplasm. After their synthesis, ribosomal proteins and assembly factors are imported into the nucleus, where they are combined with the appropriate rRNAs. Subsequently, small and large ribosomal subunits are exported into the cytosol, where they pair up to form functional ribosomes.

This Opinion Article focuses on recent findings which support the idea that, as in eukaryotes, ribosomal biogenesis in bacteria, mitochondria, and plastids is spatially organized. In these systems, there is growing evidence that rRNA processing and ribosome assembly most likely take place in association with the nucleoid.

## rRNA processing, maturation and ribosome assembly in bacteria

The processes of rRNA maturation and ribosome assembly are probably best understood in bacteria (reviewed in Kaczanowska and Rydén-Aulin, 2007; Shajani et al., 2011). In *E. coli*, the small *30S* ribosomal subunit contains 21 ribosomal proteins and a *16S* rRNA, while the large *50S* subunit consists of 33 proteins and two rRNAs, the *23S* and *5S* rRNAs (reviewed in Melnikov et al., 2012). All rRNAs are encoded in a polycistronic gene cluster and transcribed as a single precursor, which undergoes extensive processing and maturation to generate the mature rRNAs (reviewed in Deutscher, 2009; Shajani et al., 2011). The processing reactions are carried out by the exo- or endonucleolytic activities of at least five ribonucleases (RNases), including RNases III, E, G, T, and YbeY (reviewed in Deutscher, 2009; Davies et al., 2010). Furthermore, the *23S* and *16S* rRNAs undergo methylations and pseudouridylations at several positions, which are assumed to influence ribosomal structure and function (reviewed in Shajani et al., 2011).

Many of these maturation steps are coupled and take place on nascent rRNAs. The final rRNA folding pattern is probably determined by an ordered sequence of interactions with ribosomal proteins and assembly factors, which induce conformational changes and stabilize the proper rRNA structures (reviewed in Shajani et al., 2011).

It was long believed that DNA-associated bacterial ribosomes translate nascent mRNAs while these are being synthesized by the RNA polymerase (co-transcriptional translation). Striking evidence for this hypothesis came from early electron microscopy studies showing ribosome arrays (polysomes) attached to mRNA strands that were being transcribed from DNA by multiple RNA polymerase (RNAP) molecules (Miller et al., 1970). However, recent research suggests that, at least in *E. coli* and *B. subtilis*, most translation is not coupled to transcription, and that cotranscriptional translation may be limited to mRNAs encoding membrane proteins. By tracking the distribution of RNA polymerases (as markers for the nucleoid DNA) and ribosomes by means of fluorescence microscopy, a clear segregation of the nucleoid from ribosome-rich regions of the cytoplasm was observed (Lewis et al., 2000; Bakshi et al., 2012; Chai et al., 2014). Bakshi et al. (2012) found approximately 85% of the ribosomes in the ribosome-rich regions, while only 10 to 15% were detected in close proximity to the nucleoid. The majority fraction probably comprises actively translating “protein factories.” The nucleoid-associated particles are thought to be in various stages of assembly, as several rRNA maturation steps occur in a cotranscriptional and assembly-assisted manner (reviewed in Shajani et al., 2011).

Further data support the identification of the nucleoid as the site of early rRNA processing events. RNase III, which cotranscriptionally cleaves the primary rRNA transcript to yield *16S*, *23S*, and *5S* precursors, has been shown to be required for localization of the *pre*-*16S* rRNA 5′ leader region to the nucleoid (Malagon, 2013). In RNA-FISH assays for single-cell visualization, Malagon (2013) found that the nucleoid localization of the *16S* 5′ leader was dependent on the catalytic activity of RNase III, which provides indirect evidence for the presence of the enzyme in this region. Based on evidence for interplay between the Nus transcription elongation factors and RNase III in the modulation of pre-rRNA biogenesis (Bubunenko et al., 2013), Malagon further speculated that Nus proteins might serve to localize pre-rRNAs to the nucleoid.

## Localized rRNA processing and ribosome assembly in organelles

Eukaryotic plastids and mitochondria are descended from endosymbiotically acquired bacteria, and have retained their own genomes and machineries for gene expression during evolution. It is therefore not surprising that many aspects of these genetic systems, including genome organization and ribosome structure, resemble those of bacterial rather than eukaryotic systems. Similarly, evidence for sublocalization of rRNA processing and ribosome assembly events to the organellar nucleoids is now emerging.

### Mitochondria

Evidence for rRNA maturation and ribosome assembly in association with the mitochondrial nucleoid mainly derives from studies on mammalian mitochondria. Affinity purification of established protein components of the mitochondrial nucleoid results in copurification of ribosomal proteins and the ribosome assembly factor ERAL1 (He et al., 2012b). ERAL1, a GTP-binding protein with RNA-binding activity, which had previously been linked with a number of nucleoid proteins, interacts both with proteins of the small mitoribosomal subunit and its rRNA component (Dennerlein et al., 2010; Uchiumi et al., 2010). ERAL1 was proposed to act as a chaperone for the small ribosomal RNA, as its depletion leads to destabilization of the small subunit (Dennerlein et al., 2010).

Similarly, another mitoribosome assembly factor, the human GTPase NOA1 (C4orf14), a homolog of the plant Rif1 protein, has been found together with the small ribosomal subunit and translation factors in affinity-purified nucleoids (Flores-Pérez et al., 2008; He et al., 2012a). This finding prompted the suggestion that assembly of the small subunit in the mitochondrial nucleoid enables the direct transfer of newly transcribed mRNAs to the ribosome.

Like bacterial rRNAs, mammalian mitochondrial rRNAs undergo site-specific methylations, although to a lesser extent (reviewed in Rorbach and Minczuk, 2012). The enzymes responsible belong to a family of rRNA methyltransferases, some of whose members have been reported to modify nascent rRNAs in association with the nucleoid (Lee et al., 2013). A more detailed analysis of one of these methyltransferases (named RNMTL1) revealed an additional interaction between this protein and the large ribosomal subunit, suggesting that assembly of mitochondrial ribosomes begins before rRNA transcription is complete (Lee et al., 2013).

Moreover, a very recent study by Bogenhagen et al. (2014) provides evidence that mammalian mitochondrial RNA processing enzymes, like RNase P and ELAC2 (tRNaseZ_L_), as well as a number of nascent mitochondrial ribosomal proteins associate with nucleoids to initiate RNA processing and ribosome assembly. The authors therefore propose the mtDNA nucleoid as a critical control center for mitochondrial biogenesis.

### Plastids

An extensive body of evidence for localized ribosome biogenesis comes from plastids. Besides numerous ribosomal proteins, the majority of proteins with known roles in ribosome biogenesis have been identified in a comprehensive proteomic analysis of the maize chloroplast nucleoid (Majeran et al., 2012; reviewed in Germain et al., 2013). Many of these nucleoid-enriched ribosome biogenesis factors function in rRNA processing, maturation and modification, as well as in ribosome assembly (summarized in Table 1).

**Table 1:** **Nucleoid-localized proteins with proposed functions in rRNA processing, maturation and ribosome assembly in plant chloroplasts**.

**Protein**	**Protein family/domain**	**Proposed function in ribosome biogenesis**	**Maize identifier**	**Arabidopsis identifier**	**References^a^**
**PROCESSING/MATURATION OF RRNA PRECURSOR TRANSCRIPTS**					
BPG2	YqeH -GTPase	Pre-rRNA processing	GRMZM2G035042_P01	AT3G57180	Komatsu et al., 2010; Kim et al., 2012
CSP41B	Endonuclease; Rossmann fold Epimerase/dehydrogenase	Processing of 23S 1st hidden break	GRMZM2G080546_P02	AT1G09340	Beligni and Mayfield, 2008; Bollenbach et al., 2009; Qi et al., 2012
PNPase	3′ to 5′ exonuclease	23S rRNA 3′ end maturation	GRMZM2G377761_P01	AT3G03710	Walter et al., 2002
PRBP	Similarity to DEAD box helicases	Processing of 4.5S precursors	GRMZM2G436328_P01	AT2G37920	Park et al., 2011
RAP^*^	Octotricopeptide repeat (OPR) protein, RNA binding	Processing of 16S rRNA precursors	GRMZM2G050845_P01	AT2G31890	Kleinknecht et al., 2014
RBF1	Ribosome-binding factor A family protein	Processing of 16S rRNA precursors	GRMZM2G115156_P01	AT4G34730	Fristedt et al., 2014
RH39	DEAD box RNA helicase	Processing of 23S 2nd hidden break	GRMZM2G175867_P02	AT4G09730	Nishimura et al., 2010
RHON1	Rho transcription termination domain	Processing of 23S-4.5S precursors	GRMZM2G168335_P01	AT1G06190	Stoppel et al., 2012
RimM	RimM family	16S processing, ribosome assembly (in prokaryotes)	GRMZM2G165694_P01	AT5G46420	Bylund et al., 1998; Guo et al., 2013
RNase E	Endonuclease	Processing of 23S-4.5S precursor	GRMZM2G328309_P01	AT2G04270	Stoppel et al., 2012
RNase J	5′ to 3′ exonuclease and endonuclease	5′ end maturation of 16S and 23S rRNA precursors	GRMZM2G103315_P01	AT5G63420	Bollenbach et al., 2005; Sharwood et al., 2011
RNase P^*^ (PRORP1/2)	Endonuclease	5′ end maturation of tRNA precursors	GRMZM2G002642_P01	AT2G32230	Gutmann et al., 2012; Bogenhagen et al., 2014
			GRMZM2G418206_P01		
RNase Z^*^	Endonuclease	3′ end maturation of tRNA precursors	GRMZM2G125844_P01	AT2G04530	Canino et al., 2009; Bogenhagen et al., 2014
SVR1	Pseudouridine synthase	rRNA processing	GRMZM2G315806_P01	AT2G39140	Yu et al., 2008
**rRNA MODIFICATION/RIBOSOME ASSEMBLY**					
DER^*^ (EngA)	GTPase	rRNA processing/ ribosome biogenesis	GRMZM2G302233_P01	AT3G12080	Jeon et al., 2014
ERA	GTPase	Assembly of small ribosomal subunit (in prokaryotes and mitochondria)	GRMZM2G158024_P02	AT5G66470	Dennerlein et al., 2010; Uchiumi et al., 2010; He et al., 2012b
ObgC	ObgC - GTPase	Ribosome assembly	GRMZM2G077632_P01	AT5G18570	Bang et al., 2012
PFC1	rRNA methylase	16S rRNA methylation	GRMZM2G174669_P01	AT1G01860	Tokuhisa et al., 1998
RH22	DEAD RNA box helicase	Assembly of 50S ribosomal subunit	GRMZM2G100043_P01	AT1G59990	Chi et al., 2012
RH3^*^	DEAD box RNA helicase	Assembly of 50S ribosomal subunit, group II intron splicing	GRMZM2G163072_P01	AT5G26742	Asakura et al., 2012
			GRMZM2G415491_P01		
RIF1^*^ (NOS1, NOA1)	GTPase (YqeH)	Ribosome assembly	GRMZM2G384293_P01	AT3G47450	Flores-Pérez et al., 2008; Liu et al., 2010
RsmD	rRNA methylase	16S rRNA methylation (in prokaryotes)	GRMZM2G010801_P01	AT3G28460	Lesnyak et al., 2007

*All proteins listed were identified as nucleoid proteins by Majeran et al. (2012). Proteins known to be involved in ribosome biogenesis but not identified in their nucleoid fraction, as well as factors that probably have only indirect effects on rRNA maturation and ribosome assembly, are not included in this list*.

* *Nucleoid localization demonstrated by groups other than (Majeran et al., 2012) (see text)*.

a *Only most relevant publications referring to a function in ribosome biogenesis or nucleoid localization are listed*.

Among the processing and splicing factors identified are many plant homologs of bacterial RNases that have been reported to be responsible for exo- and endonucleolytic cleavage of the large rRNA precursor and maturation of cotranscribed tRNAs (reviewed in Stoppel and Meurer, 2012; Germain et al., 2013). Most of the nucleoid-enriched proteins involved in ribosome assembly and rRNA modification are classified as either GTPases, RNA helicases or rRNA methylases (Table 1).

Two of the nucleoid-enriched plastid proteins listed in Table 1 are homologous to the mitochondrial enzymes ERAL1 and NOA1, for which a nucleoid localization has also been reported (see above). For several others, links with the nucleoid are further supported by alternative approaches as exemplified in the following.

An immunological analysis of maize chloroplast subfractions revealed that the DEAD-box RNA helicase RH3, thought to function in assembly of the *50S* subunit, localizes to the chloroplast stroma and thylakoids, as well as to nucleoids (Asakura et al., 2012). For two recently characterized plant proteins required for ribosome biogenesis, DER and RAP, cytological evidence for nucleoid localization comes from GFP fusion experiments (Jeon et al., 2014; Kleinknecht et al., 2014). DER is a Double Era-like GTPase, whose bacterial homolog (also known as EngA) acts as a ribosome assembly factor in *E. coli*, was found to bind to the *50S* ribosomal subunit and to play a role in pre-rRNA processing in tobacco (Hwang and Inouye, 2006; Jeon et al., 2014). The *Arabidopsis* protein RAP is a member of the Octotricopeptide Repeat (OPR) protein family, and binds to the 5′ leader sequence of the *16S* rRNA precursor (Kleinknecht et al., 2014). Depletion of RAP specifically affected the trimming/processing of the chloroplast *16S* rRNA precursor, which supports the identification of the nucleoid as the site of *16S* rRNA processing in chloroplasts. Preliminary data imply that RAP has no intrinsic RNase activity, and might influence *16S* rRNA maturation by conferring sequence specificity on an RNase or by modulating RNA secondary structures to control the accessibility of an RNase recognition site within the *16S* precursor (Kleinknecht et al., unpublished results).

However, in the case of many chloroplast proteins reported to be involved in various steps of ribosome biogenesis, GFP-based fusion studies provide no support for specific localization to the plastid nucleoid. One possible reason for this may be the use of transit peptide-GFP instead of full-length protein fusions, as determinants of nucleoid localization are unlikely to be encoded in the transit peptide. Accordingly, Park et al. (2011) also reported the localization of the protein PRBP, which likely functions in *4.5S* rRNA processing, to distinct spots within chloroplasts, which probably represent nucleoids. This sublocalization was only observed when the full-length protein was fused to GFP and not with a transit peptide-GFP fusion. Nevertheless, other proteins for which full length-GFP fusions were used could not be clearly assigned to the nucleoid (e.g., Flores-Pérez et al., 2008; Yu et al., 2008; Chi et al., 2012). This might be due to weak or transient association of the respective protein with the nucleoid. Alternatively, some of these proteins may perform further functions in other organellar subcompartments leading to ambiguous localization signals.

## Conclusion and future perspectives

Localization of rRNA processing and ribosome assembly to organellar nucleoids seems to be a general phenomenon derived from a bacterial ancestor. Unlike the case of the eukaryotic nucleus, no physical barrier intervenes between bacterial/organellar RNA synthesis and translation. The nucleoid might therefore provide a scaffold for an intra-organellar microenvironment which enables coupling of rRNA transcription to ribosome assembly. This might not only enhance the efficiency of ribosome assembly by substrate channeling, but also largely prevent the precocious association of mRNAs with immature *30S* ribosomal subunits. However, it remains to be shown whether the colocalization of rRNA processing and ribosome assembly with nucleoids has functional significance or simply reflects the rapid kinetics with which ribosome biogenesis factors bind to nascent rRNA targets.

Nonetheless, the growing awareness of the requirement for sublocalized cellular processes in apparently less organized bacteria and organelles, and the availability of more sensitive detection techniques including super-resolution imaging, will undoubtedly lead to new insights into the spatiotemporal organization of ribosome biogenesis in the future.

### Conflict of interest statement

The author declares that the research was conducted in the absence of any commercial or financial relationships that could be construed as a potential conflict of interest.
